# Potential Barriers to Participating in Cancer Moonshot Biobank for Low-Income Patients with Cancer of Rural Maine

**DOI:** 10.1177/19475535251391568

**Published:** 2025-11-13

**Authors:** Mike Kohut, Jamie Saunders, Neil Korsen, Anne Breggia, Jill Prescott, Scot C. Remick, Susan Miesfeldt

**Affiliations:** 1 MaineHealth Institute for Research, Scarborough, Maine, USA.; 2 MaineHealth Cancer Care Network, Scarborough, Maine, USA.; 3 Tufts University School of Medicine, Boston, Massachusetts, USA.

**Keywords:** biobanks, patient perspectives, biopsies, qualitative, genomic tumor sequencing

## Abstract

**Background:**

The National Cancer Institute’s Cancer Moonshot Biobank (CMB) aims to accelerate research on tumor sensitivity and resistance to standard-of-care therapies through collection and distribution of longitudinal biospecimens donated by research participants with cancer. Since low participation among historically underserved populations limits the generalizability of research done with biospecimens, CMB supports local community engagement activities.

**Objectives:**

We assessed the factors for enrollment in CMB and related precision oncology trials among rural, low-income adults with cancer who are served by the multi-site MaineHealth Cancer Care Network (MHCCN). We sought to address barriers thus identified.

**Methods:**

From October 2021 to May 2022, semi-structured interviews with MHCCN clinical research coordinators (4), oncologists (6), and patients (15) elicited perceived facilitators and barriers to participating in CMB and related trials for low-income rural Mainers. We developed a descriptive model of the steps by which patients become CMB participants based on reports from research coordinators. Factors impacting recruitment were identified at each step.

**Results:**

Rural clinics have limited staff to monitor patient lists and collect samples. Many oncologists were skeptical of clinical benefit and correspondingly reluctant to recruit vulnerable patients. Patients were generally open to CMB if recommended by their oncologist but expressed concerns that involvement in CMB or related research would consume limited time, lead to another biopsy, or threaten privacy.

**Conclusion:**

Addressing barriers for low-income, rural residents improves access for everyone. To reduce staff burden within a health system, better resourced sites can provide infrastructure and personnel support to sites with fewer resources. Education may correct misunderstandings and improve awareness of the benefits of CMB and related research involvement among research staff, oncologists, and patients.

## Introduction

The National Cancer Institute’s (NCI) Cancer Moonshot Biobank (CMB) seeks to create a resource for investigation of the mechanisms of tumor sensitivity and resistance to standard of care therapies across a spectrum of tumor types. Through NCI Community Oncology Research Program (NCORP) and National Clinical Trials Network member sites, tumor biospecimens, and associated data are collected longitudinally from individuals with advanced cancers, receiving standard-of-care therapy, including targeted therapy.^1^ Participants donating fresh biopsies receive no-cost tumor genomic reports for their providers. While individuals can enroll by donating archival tissue, they do not receive a genomic report. CMB is not a clinical trial, though the tumor genomic report may reveal drug trial eligibility.^1^

CMB has provided resources to study sites to ensure that all eligible persons, including those facing geographic, socioeconomic, and other barriers, have access to emerging cancer prevention and control activities, and opportunities to participate in cutting-edge clinical research.^2^ Thanks to such support, we studied the barriers and facilitators to enrollment in CMB and related biobanking and precision oncology trials in the MaineHealth Cancer Care Network (MHCCN), an NCORP site that included, at time of study, 11 member hospital organizations, including Maine Medical Center-Portland. MHCCN serves 11 of 16 Maine counties and offers direct care to nearly 70% of Maine’s cancer population annually.

In order to most effectively identify and address the biggest participation barriers, we focused on people most likely to experience barriers in Maine—those with low income and living in rural areas. Sixty-one percent of Maine’ s population is rural, higher than every other state except Vermont.^3^ Given the structural vulnerabilities associated with rurality, many people in Maine are likely to face barriers to participating in research.^4-6^ Rural residents have fewer opportunities and face greater obstacles to participate in biobanking and cancer research than their urban peers.^7,8^ Rural inhabitants with fewer financial resources will have additional barriers to research participation.^9,10^ Our health equity approach, based in the principle of “centering the margins,” contends that addressing barriers for the most marginalized will address barriers for everyone.^11^

## Materials and Methods

As our goals were exploratory in nature, we adopted methods to maximize discovery of relevant information. Our analytical approach permitted inductive coding of barriers and facilitators. As we wanted to ensure participants could provide information not anticipated by researchers, we used semi-structured interviews, which afford the interviewer flexibility to probe additional topics introduced by participants. We collected and triangulated perspectives from three types of participants who play key roles in the process of cancer-affected adults becoming research participants—clinical research coordinators (CRCs), oncologists and patients themselves. Our analysis established first how individuals in Maine come to participate in CMB, and second which specific enrollment barriers or facilitators that rural and low-income individuals face.

The MaineHealth Institute for Research IRB reviewed and approved this study, including study design and patient recruitment. All participants gave verbal informed consent prior to interviews and received $40 gift cards following participation.

### Sampling

**FIG. 1.**
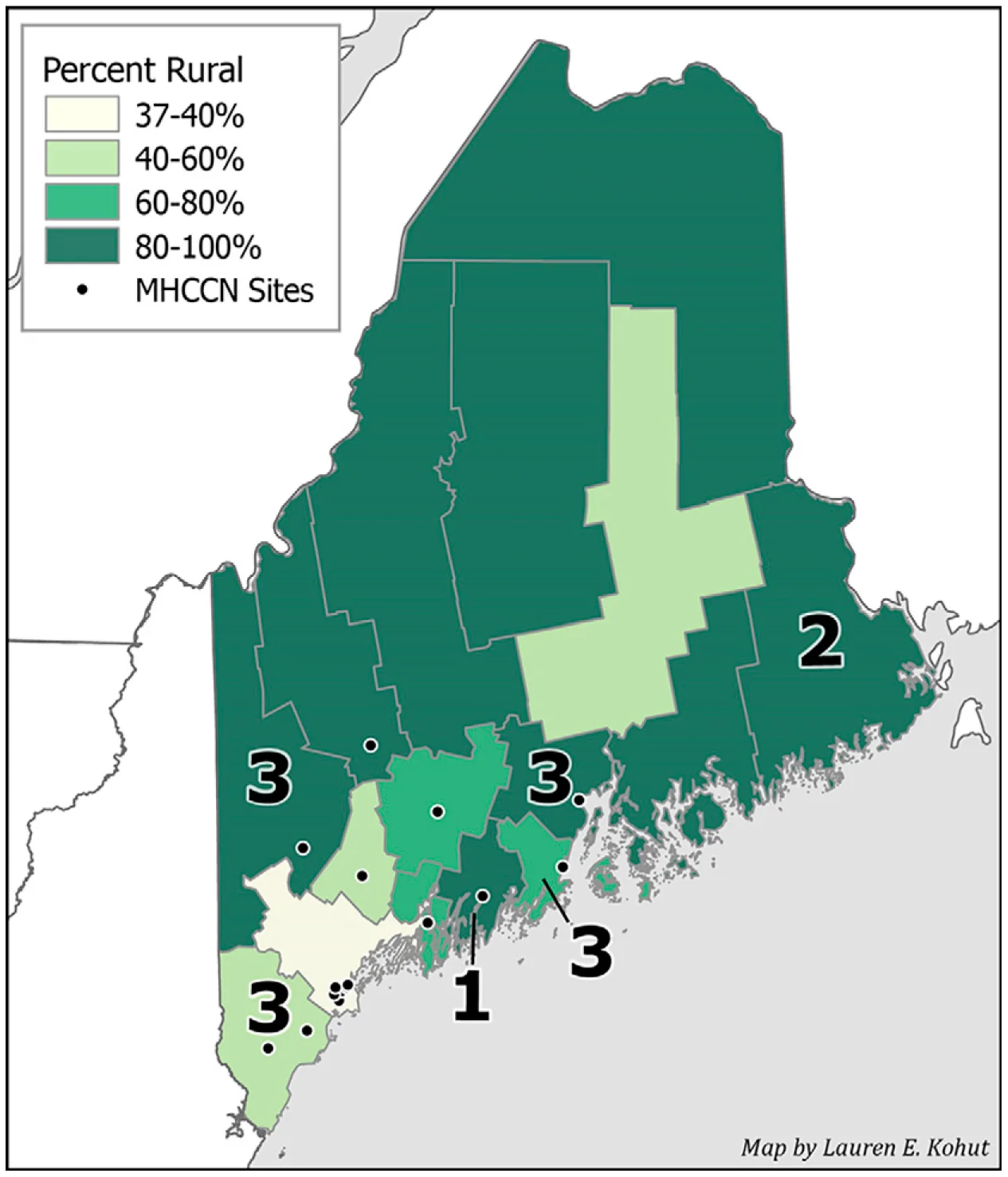
Counties in Maine by the percentage of the population living in a rural area, as defined by the U.S. Census. MaineHealth Cancer Care Network (MHCCN) sites that can participate in the Cancer Moonshot Biobank are shown by location. Arabic numerals indicate the number of patient participants in the sample living in each county. Created by Lauren E. Kohut.

We used purposive sampling^12^ focused on three groups likely to encounter or witness barriers to biobanking and cancer research participation in rural Maine—residents with limited income and a cancer diagnosis, and the oncologists and CRCs who may provide them care. Working with rural-facing cancer care sites and cancer advocacy organizations, we recruited in or near non-metropolitan counties, defined by the Office of Management and Budget (OMB) as containing no urbanized area with >50,000 residents (see Fig. 1: Map). At the time of the study, 12 CRCs supported rural-facing MHCCN clinics and 16 MaineHealth oncologists were serving substantial rural populations. All CRCs and oncologists working at these sites were invited by email to participate.

Patient participants were recruited through flyers and word-of-mouth from advocacy organizations and clinical staff, including social workers, and instructed to contact us to schedule the interview. We also traveled to two rural clinics and conducted face-to-face interviews with individuals there.^13^ Patient interviewees were not enrolled in CMB at time of interview, as this qualitative study is separate from the actual CMB study. Accordingly, patients could only answer hypotheticals about anticipated barriers or concerns, though their responses were informed by lived experience with cancer.

Patients were eligible for interview if they had been diagnosed with any type of cancer, excluding non-melanoma skin cancer, within the previous 5 years, were over age 18 years, and able to understand the study and give verbal consent. To protect privacy, eligibility screening did not include income or county of residence. We recruited patients until thematic saturation, when additional interviews were no longer producing new insights relevant to our research aims.^14^

### Data collection

All interviews were conducted one-on-one by co-author M.K. during Fall 2021 and Spring 2022. The interviewer had extensive qualitative research experience, including on topics related to tumor genomics.^15^ Interview guides were tailored for each participant type, reflecting knowledge, and experience of that role (included as Supplementary Data S1 and S2). Interviews with CRCs and oncologists occurred via Zoom. CRCs reported challenges they and patients experienced. Oncologists described the value they ascribed participation, decisions to tell patients about CMB, and concerns heard from patients and staff.

M.K. interviewed 12 patients in-person during visits for cancer treatment, and 7 via telephone. Interviews with patients elicited experiences with and perspectives on biobanking, biomedical research, genomic tumor testing, and biopsies—reflecting different components of CMB. We asked about types of research relevant to CMB, including cancer research in general; cancer clinical trials; cancer genomic research; and tumor biobanks. Anticipating that many patient participants would be unfamiliar with cancer genomics, we read the following description before asking about it:

Cancer genomics is where researchers compare the genes in normal cells in your body with the genes of cancer cells in your body. They look for the differences between those genes, and those differences could be useful for figuring out how the cancer will respond to different kinds of treatments.

Participants shared concerns about these types of research, challenges anticipated with participation, and reasons they may or may not enroll. All interviews were recorded and professionally transcribed.

### Analysis

We began our analysis by indexing transcripts by topic, using the software MAXQDA^™^ (Version 22.8.0) to manually attach codes to segments of the transcripts. After identifying excerpts relevant to specific topics, we sub-coded those excerpts inductively.^16^ Analysts identified phenomena reported to impact CMB participation by making it more difficult/less likely (barriers) or easier/more likely (facilitators). As barriers and facilitators were frequently inversions of one another, we present our results in terms of the factors within which barriers and facilitators impact participation.

We employed a variety of practices to enhance the quality and credibility of our results. Interpretive claims were restricted to interview text; alternative interpretations were sought and considered. Analysts adopted a hermeneutics of faith to interpret interview responses—understood as trusting participants to share their perspectives honestly, while also recognizing that each perspective is limited and may misunderstand aspects of the whole.^17^ We triangulated what we heard from our three types of participants to identify relevant factors impacting different parts of the recruitment process. Our team presented those factors identified to CRCs and a patient advisory panel for feedback.^18^ Both groups confirmed findings were consistent with their experience.

## Results

### Participant characteristics

All patient participants reported living in a county defined by the U.S. OMB as rural (see Fig. 1). We excluded from our analysis four individuals who reported annual incomes over $75,000 (U.S. median income). We included six patient participants who declined to share income but reported not having a college degree, as the association between education attainment and income is well-established.^19^ The sample reflected a broad range of cancers. Most patient participants (11/15; 73%) reported having received active treatment within the past year. Patient participant characteristics are summarized in Table 1.

In addition to these patient participants, 6 oncologists (38% of total invited) and 4 CRCs (33% of total invited) were interviewed between February and May 2022. We believe that thematic saturation was reached because responses were stable and consistent across interviews, with no additional themes after the third interview.^14^ This size of our sample is consistent with research on adequate sampling for this type of analysis.^20,21^ Oncologist and CRC participants were asked about experience with CMB and other precision oncology research. They also estimated the proportion of their patients who were low-income and rural. All these participants reported working with mostly rural patients for more than a year, and all worked at MHCCN sites where CMB recruitment was possible. Additional characteristics of these non-patient participants are shown in Table 2.

### Factors impacting CMB recruitment/enrollment process

**FIG. 2.**
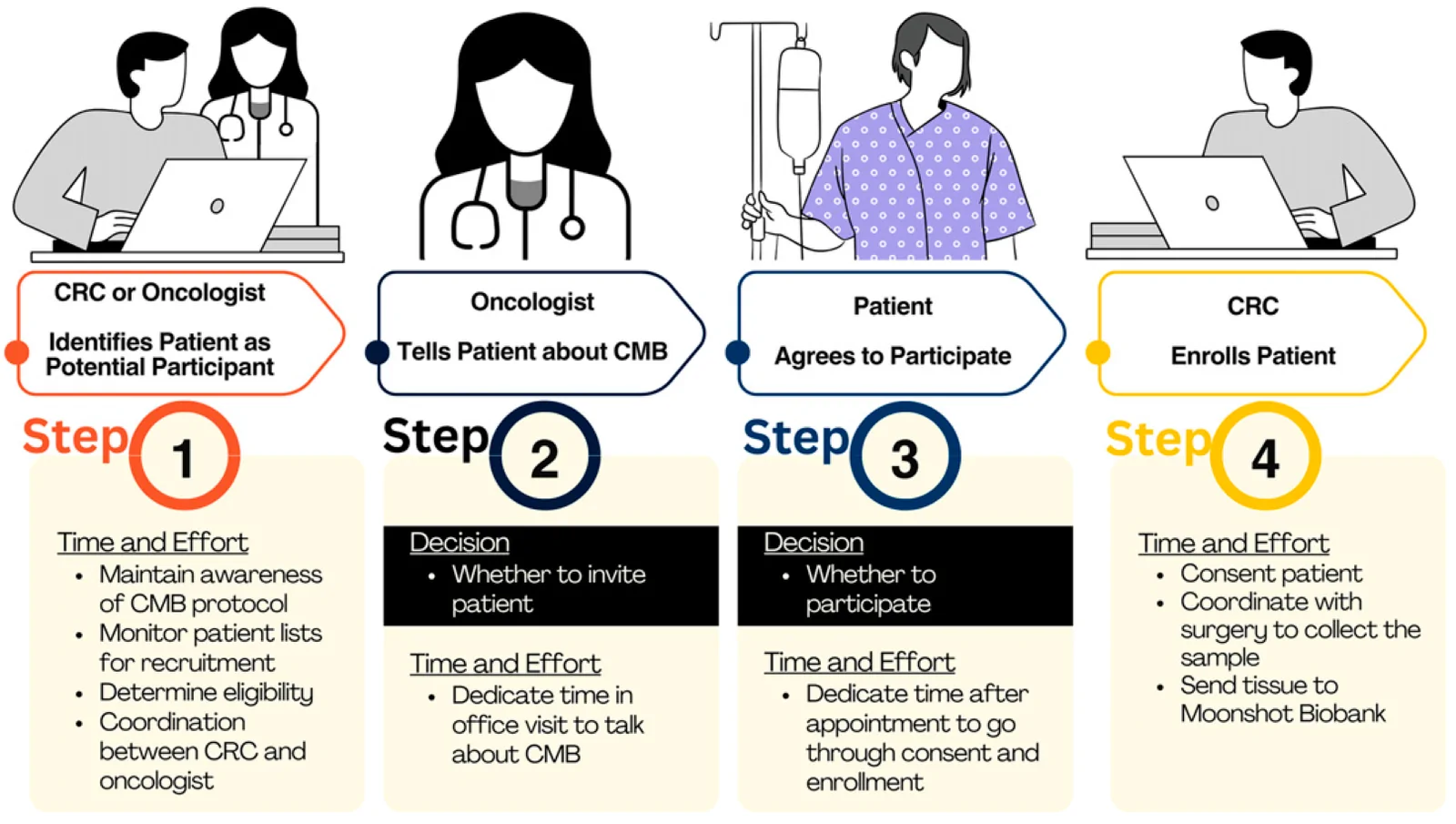
Descriptive Model of CMB Patient Enrollment. Decision points are noted with a dark background. Required time and effort is noted with a light background.

Based primarily on interviews with CRCs, we mapped out a descriptive model of the steps leading to CMB enrollment, recognizing that this process requires cooperation among individuals serving different roles (Fig. 2). Based on interviews from all three participant types, we identified factors impacting enrollment, related to decisions and the time and effort involved in each step. All reported findings are grounded in statements from at least three independent participants to limit idiosyncrasy. We chose not to report exact frequencies of responses since these numbers could be misleading given the small, non-representative sample. Where deemed relevant, we used indefinite quantifiers of frequency. Organization of results is based on the four steps outlined in Figure 2.

## Identifying Patients as Potential Participants

In the first step, either a CRC or oncologist identifies CMB-eligible patients, by monitoring patient lists for clinical characteristics, cancer progression or scheduled biopsy. While patients could theoretically initiate enrollment themselves by asking the oncologist, none of our participants gave examples of this. Instead, enrollment was either initiated by an oncologist, based on possible clinical benefit, or by a CRC at the treatment site, based on eligibility, including consideration of the following:

### Timing of biopsy

Asked what patient attributes make them good candidates for CMB, most oncologists said they thought about CMB when a biopsy was planned:

I’ve had a couple of patients where they were going to have a biopsy anyway, were having surgery, and it just happens that we needed, we were going to send tissue anyway for genomic research or genomic studies commercially. And we thought, “Well, this is free.” The readout from NIH is actually pretty good and pretty comprehensive. So it’s a win-win.

(Oncologist 3)

These situations were relatively rare, however, as many patients already have had a biopsy before seeing an oncologist:

… sometimes we don’t see patients before they have surgery. Sometimes we just get patients via post-op. So the opportunity has passed at that point to enroll them.

(CRC 3)

Enrolling a patient prior to their first biopsy is further complicated by the state of mind of the patient, who may be overwhelmed. One CRC recalled another study when reflecting on this:

… they’re getting bombarded with surgery consult, oncology consult, meeting with their navigator, social worker consult, maybe financial consults. Then they ask me to come in, by the time I get there, they’re like, “I can’t think anymore.” So then I tell them, “Oh, we just need these specimens at these times.” And they’re like, “No, too much right now.” That was the biggest barrier with that study.

(CRC 2)

In several cases where cancer progression prompted a tissue biopsy or surgical resection for prognosis and/or treatment, the oncologist used CMB to access a genomic report for treatment decision making:

Those sorts of patients who saw us for early-stage cancers and then progressed, they’ll still be on our radar. And then for those patients, usually we are the ones ordering the biopsy, so they’re easier to capture for Moonshot.

(Oncologist 2)

Concerns about biopsies were reduced in cases where they could be performed less invasively, as for skin lesions, or otherwise more accessible tumors. One oncologist said the ideal candidate is:

Someone who is younger, doesn’t have too many comorbidities, is eager to help, and has accessible tumor like a lymph node somewhere where like, let’s say, the neck, or with skin lesion, or somewhere where very invasive procedure is not needed like liver or lung, retroperitoneal lymph node.

(Oncologist 4)

### Monitoring capacity

Due to competing demands, few oncologists regularly monitored their patient lists for CMB eligibility. In contrast, CRCs monitor patients for eligibility in all studies as part of their role. However, in rural clinics with limited staff, nurses, and coordinators operate with the time available:

We screen patients the day maybe before their visit and identify; and then we’ll tell the physician; and he’ll introduce the study; and then we’ll go down and talk to the patient about it. If the day before we haven’t had the opportunity to screen the next day’s patients, then that doesn’t occur. […] Then maybe we get them on the next cycle or the next clinic visit or whatever. But sometimes all the patients don’t get screened for enrollment to clinical trials just for lack of time.

(CRC 3)

Without the time and ability to monitor patient lists, patients may not be identified as eligible.

## Telling Patients about CMB

After an eligible patient has been identified, the second step involves the oncologist’s decision about whether to discuss CMB with the patient. Oncologists have limited time and weigh different priorities in deciding whether to discuss CMB with patients:

And obviously we take the path of least resistance a lot. When we’re really pressed for time, I do not have time to spend 30 extra minutes talking about a clinical trial. And, that happens a lot. There’s no question. […] it’s like so many things during your day block you from really being able to move forward. Or the patient can’t hear, or they’re not feeling good that day, or the wife didn’t come in with the husband, and we really need the wife there because he doesn’t want to make any decisions without her participating in it. There’s so many logistics, things that stop us.

(Oncologist 6)

Oncologists consider a range of factors, specific to patients, studies, and clinic capacity in making this decision, including:

### Oncologist understandings of CMB

Oncologists were universally familiar with different kinds of research, and the majority readily acknowledged the value of research for advancing medicine, speaking approvingly of the scientific merits of CMB and related research:

I’m all for it. And this is where the future of cancer is, it’s targeted therapies based on tumor genome testing. And I think we should be devoting more resources into studying and developing therapies based on that. So I think it’s extremely important.

(Oncologist 2)

However, some oncologists did not consider patients for CMB due to the inaccurate assumption at the time of this work that patients could not be enrolled with archival tissue. Several thought that participation required patients to get research-only biopsies, which they saw as too high a burden:

We’re going to do a biopsy and every year, every two years on you, is that okay? Tumor could be gone, it could be invasive, it could be painful, it could be at risk, people moved. Sure. Be a lot of problems with that.

(Oncologist 1)

The CMB protocol does include optional research-only biopsies after cancer progression; however, this is not repeated on a scheduled basis. Another area of uncertainty related to the cost of study-related biopsies or genomic testing. Oncologists less familiar with the study questioned whether there were hidden patient costs:

I, as a provider, don’t have knowledge of, like, how much is it going to cost them because different insurances are different. […] I mean, they are hidden because they’re so insurance driven. Some insurances would cover it, some insurances don’t cover it. If there was like a known cost that, “Okay, this test is only going to cost you $20 no matter what kind of insurance you have.” Then it’s easier for me to propose it to patients and be like, “It’s not going to cost you more than what you anticipate.”

(Oncologist 4)

### Anticipated clinical benefit

All oncologists said they were more willing to talk to a patient about research when they anticipated a clinical benefit to the patient. The clearest potential benefit of participating in CMB is obtaining a genomic report that may support treatment decision-making.

It was actually very beneficial to enroll the patient because we got the report, and it showed us some excellent targets that would help the patient.

(Oncologist 2)

Many oncologists voiced desires to order tumor genomic testing for all patients to identify new treatment options, e.g., off-label drugs, or promising clinical trials:

… if we can get any information that leads to an option for therapy that we didn’t otherwise know we had, that’s great. Although I haven’t found these therapies to work that often, but patients really look forward to having another option available, and we all do. […] we’ve had some cases and know it made a huge difference and really gave them a therapy that was highly successful.

(Oncologist 6)

While tumor genomic testing is increasingly covered by insurance and available through private companies, some patients may save costs through a study. Oncologists told us they were more likely to suggest participation in CMB if it is the best and easiest pathway for genomic testing and timely reporting of results. They were correspondingly less motivated to tell patients about CMB in the absence of this benefit:

If we don’t get the [genomic report] then I don’t have anything to tell the patient. […] Because if you tell the patient, “Hey, I want to use your tissue, which we have. And if we need a report of how this can help you, we might need to do another biopsy.” I think majority of my patients are not going to agree to that.

(Oncologist 4)

Many oncologists pointed out they already had access to genomic reports through a commercial source and were thus less willing to slow down the process or burden patients by using CMB instead.

### Perceived interest from patient

Oncologists often mentally “pre-screen” patients based on their perception of a patient’s openness to participate in studies like CMB. They are sensitive to each patient’s cancer experience and may avoid bringing up a study while patients are still processing a recent diagnosis or evidence of progressive disease.

“… sometimes patients are just too overwhelmed and then you sort of lose the window because you’re getting a biopsy and at that point, the patient has not given consent. So you lose that window at that point to enroll the patient. Because then, as I said, archival tissue is not accepted.”

(Oncologist 2)

Likewise, there was a sense among some oncologists that some patients in rural areas are simply not interested in research:

I think these patients are just, culturally anyway, they don’t feel invested in the academic world. […] my pitch with them is always, “It’s patients who let us do this kind of work that have taught us everything we know about cancer. And what we have to offer you today is because of patients who did that. So wouldn’t you like to participate?” But they’ll say, “Nah.” I get that a lot. So yeah.

(Oncologist 6)

### Consideration of burden to patient

Oncologists were clear that they are primarily concerned with caring for their patients and are hesitant to cause them any additional stress, particularly in situations where research provides no clinical benefit. Oncologists reported that they are less apt to encourage research participation that involves extra travel or patient time, including completion of surveys.

I think it just depends on what the ask is for patients. I mean, if the patients have to make special trips to sign the consent, or to do multiple blood draws. […] I think you have to try not to make it two hours for people to do the follow up stuff with surveys […] when people come to a doctor, often, they may or may not be driving. They have to bring someone with them. The other person may be working, has to take time off from work. There’s a lot of kind of logistics under the radar that we don’t necessarily understand.

(Oncologist 3)

More than anything else, coordinators and oncologists cited the burden on patients of extra biopsies:

I’ve seen my patients be more open to like blood specimens. Anytime you talk to them about taking more tumor outward or doing a special procedure to be able to get fresh tissue just for a study, they would not consider that. Even in the name of research, even in the name of getting more information.

(Oncologist 4)

### Consideration of burden to office/staff

Oncologists considered burdens to the clinic and research staff, particularly since smaller, rural clinics already experience high staff demands.

… with the Moonshot I was a little bit surprised that they don’t accept archival tissue for testing. You have to have fresh biopsies that have to be sent over. That’s a little bit of a burden on our research staff at times. Like the patient who I enrolled was having a surgery and my research nurse had to spend like six hours in the OR to collect the tissue, which is not feasible at all times.

(Oncologist 2)

## Agreeing to Participate

The third step in the process is when an individual decides whether to participate. Participants noted it was difficult to predict how the cancer experience will impact attitudes toward research participation among patients. Cancer is deeply personal and can affect a person in unexpected ways. For some, participation in research is an opportunity to give back and ensure something positive will come from their illness, while others reported feeling more protective of themselves. Aside from such personal considerations, patient participants cited several factors as shaping their decisions to participate:

### Recommendation from oncologist

Over half of patient participants said they expected or preferred to hear about promising research from their doctor. For several, the most critical factor in whether they would agree to participate in a biobanking study is their oncologist’s input. While it is expected that patients with more education and resources may learn about studies independently, rural residents with lower incomes may rely more on information about research from their oncologists:

But what’s interesting to me in my population, which may be different than other populations, was I rarely had patients that knew anything about it before I started to talk about it, or who had seen it on the internet and really were asking for it. It wasn’t very common.

(Oncologist 6)

Patient participants generally agreed that, unless their oncologist tells them about a study, they are unlikely to know about it:

We only can go by what our oncologist tells us. […] When people have cancer, you just have to put all your faith in that doctor.

(Patient 4)

Further, without an explicit recommendation from the oncologist, several patient participants said they would be hesitant to enroll:

… because [my oncologist has] been through it and he maybe picks up different things that maybe they’re looking for […] I’d feel more comfortable if somebody in his positions would say, “It’s a good idea.”

(Patient 10)

Overwhelmed with complicated information and treatment options, many expressed a willingness to do whatever their oncologist recommended.

### Understanding of cancer research

Patient decisions about enrolling were shaped by their understanding of research. In interviews, patient participants shared perspectives on cancer research in general, as well as on clinical trials, genomic research, and biobanks. Overall, they recognized many ways that research could be valuable, both personally and for others. Most participants agreed that research is important to medical advancement, and several claimed altruistic motivations for participation:

By being in research that maybe somebody else will benefit from the research that I’ve been involved in. If I can’t benefit from it, I want somebody else to be able to benefit from it.

(Patient 4)

Familiarity with biomedical research, through education, employment, or personal experience, often predicated positive assessments of research. Participants less familiar with research raised more concerns. One participant wondered whether participating in a study would result in their contact information being sold for profit:

Because people do things, companies are doing it for money. Just like [organization], for example, they sell their mailing list to other companies, but they shouldn’t do. Same with other companies and they don’t ask you if they can sell your name on the mailing list. You just have to be careful who’s doing it and who’s in charge.

(Participant 10)

Decisions to enroll require trust in the study sponsor. Asked whether it mattered to them whether research was funded by corporations or the government, around half expressed no preference. Others registered concerns about the profit-motives in researchers, but such concerns were not necessarily limited to privately-funded research:

I think they’re all the same now. […] it really gripes me to see so much federal money go into so many of these universities and research labs and then who walks away with the [profit]?

(Patient 1)

Several participants even expressed more concern about government involvement than for-profit entities:

Patient 6: Government kind of concerns me right now, the way the world’s going. So I don’t have much faith in them.

Interviewer: Okay. So if it were being sent to government researchers, you might be concerned?

Patient 6: Yeah.

Interviewer: And then what about a private pharmaceutical company?

Patient 6: That wouldn’t bother me.

### Awareness of types of cancer research

Patient participants shared perspectives on different types of cancer research, including understandings, concerns about participation, and reasons to enroll in each. Table 3 provides a summary of participants’ [mis-]understandings, concerns about participation and reasons to participate in different kinds of cancer research (see Table 3). When asked about “research,” most participants understood it to specifically reference therapeutic trials of “experimental” drugs:

It’s a trial, like it’s not approved. […] I guess that would take more than a little interview to know if that is something I would do or not.

(Patient 13)

What do you think I think? I think you’re a test bunny. That’s what I think, like a rat in a cage.

(Patient 11)

While many acknowledged likely side effects, they nevertheless saw clinical trials as an opportunity to access promising agents:

Well, they’re trying out drug that they believe will work, and you’re getting a chance to use it before it’s out on the market, basically.

(Patient 8)

### Genomic research

Only two participants were familiar with genomic research. Many conflated it with genetic tests:

I guess […] when they can figure out or have figured out that certain types of genes perhaps that come down through the family is potentially a cause for cancer down the road.

(Patient 2)

After hearing our definition of cancer genomics, most said that this kind of research would lead to better treatments. However, comments about these hoped-for treatments suggested over-estimation of benefits. They imagined treatments would be programmed to “target” cancer cells while not impacting healthy cells:

I would hope that having that particular genome pattern for that particular cancer, there would be a particular cure. […] I mean, look at chemo, chemo hits the whole body, radiation affects all surrounding areas and just there’s so many side effects, whereas I think this is so targeted that it’s going to cure without damaging.

(Patient 1)

While such over-estimation could facilitate enrollment, expectations of a targeted cure may be inaccurate.

### Biobanking

In contrast to therapeutic trials, most participants understood biobanking to be less risky or even without risk. Several said they assumed their excised tissues were already being used for research. Asked about donating tissue to a biobank one participant responded:

It wouldn’t bother me at all because once [my tissue]’s gone, once they take it, […] then after that you shouldn’t really think about it because what’s there to worry about after they do it? Unless they’re using it for other than what they said they would use it for.

(Patient 10)

### Burden of participation

Individuals already overwhelmed with cancer may be more protective of their time and bodies, driving research participation hesitance:

There’s a lot of appointments, there’s a lot of time being consumed when you are going through treatments. So, if it could be more convenient […] especially in a rural area where it’s not just five minutes to get to the place, it’s 45 minutes…

(Patient 13).

While most patients interviewed reported being open to research, participation costs and efforts made enrollment less attractive. Few participants were willing to travel long distances for research. A number of clinical trials for people in Maine are accessed in Boston, which was a 5-hour drive for some participants. Participants especially reported wariness around studies requesting research-only biopsies, which can require travel and other inconveniences and are painful:

They just stick this long needle in there and the first one I had, I knew it was going to hurt. I saw the needle, which was stupid. I wish they’d had it covered when I went in the room. I had a washcloth in my mouth and I knew, and my husband said I still screamed so loud the whole hospital probably heard me.

(Patient 6)

Notably, participants reported no concerns related to liquid biopsies. Samples of blood can be obtained more easily, at a negligible burden.

### Prospects for treatment

Many patients were understandably focused on personal clinical benefit. Attitudes about participating in research sometimes depended on whether a patient participant was satisfied with their current cancer treatment and the availability of other options. Some, perhaps assuming research always entails therapeutic trials, were uninterested in participating in any research if their clinical course was stable:

At this time, I don’t need that. There’s people that definitely need that because of their cancer. I don’t know what the word is—chances of death are greatly increased. You know what I’m saying?

(Patient 7)

In cases where treatment has failed, individuals suggested greater willingness to participate in research that could lead to other treatment options:

I guess if […] the cancer was growing and I was desperate. I guess I would certainly jump in because I can’t see damage, I don’t see the damage in it. I don’t see where it would hurt you.

(Patient 1)

One participant with stage IV cancer suggested they would participate in research, even if it required driving a distance, but only:

As long as I was getting some kind of treatment. I don’t want to go down there and not get treatment, right?

(Patient 9)

## Enrolling Patients

If a patient agrees to participate, the fourth step is the actual enrollment. The patient meets with a CRC who explains the study, obtains consent, and facilitates CMB enrollment and tissue collection and handling. The consent process takes approximately 30 minutes and is accomplished either using hardcopy documents reviewed by the CRC prior to patient signature or an eConsent process, whereby the patient watches a short video, answers teach back questions, reviews the informed consent and signs the documentation on a CMB-provided tablet. We identified no barriers for consent, but biospecimen collection was reported as a significant burden on rural-facing clinics.

### Time to collect samples

An important barrier to participation was the very substantial time required of research staff to collect fresh tumor samples. CRCs reported spending hours waiting through surgery for fresh biopsies that meet CMB requirements. At smaller clinics, coordinators may not have time to engage with many studies. In the case of CMB, the burden of specimen collection can be a limiting factor:

I know patients who just need say a simple EKG, they’re scheduling like three or four weeks out because every department is so short staffed. […] I’m drawing blood in the morning for the lab because they have no phlebotomist. There are just no staff to do anything. And then to ask them to do a procedure which would take three staff members and three or four hours out of their day for an unnecessary procedure, I could see there being a lot of pushback.

(CRC 1)

As previously noted, oncologists may anticipate this burden on staff and decide against inviting patients.

## Discussion

CRCs, oncologists, and patients in rural Maine described several important factors impacting recruitment for CMB, which may be relevant to similar projects and comparable patient populations. Rural clinics often have limited available research staff to consistently monitor patient lists and carry out demanding study protocols. Rural patients may have misconceptions about research, and be skeptical of it, especially if researchers are perceived to be profit-motivated. With fewer financial resources, patients may find any demands on time and effort unduly burdensome. Studies that collect tissue biopsies were seen as especially burdensome, potentially involving cost, pain, and travel. If a patient believes a study may involve changing treatment, then patient interest is contingent on satisfaction with current treatment. Ultimately, our results suggest that, for CMB and other biobanking, the biggest factors were not about patient decisions to participate, but oncologist decisions to tell patients about the research and recommend their participation. Patients look to their oncologists for guidance, and their recommendations are correspondingly key to successful recruitment. It is therefore critical to note that many oncologists are reluctant to tell patients about CMB and other precision oncology research if they do not see a clear clinical benefit of participation or if the related burden seems too onerous.

While our interviews focused on CMB, our findings are mostly consistent with other research on barriers to precision oncology research and biobanking,^23^ though we note several differences between our participants and those living in urban areas. Our results are more consistent with other studies of rural individuals, which report they may be skeptical about how their data will be used,^24,25^ suspicious of exploitation,^22^ or concerned about the burden of participation.^22^ A systematic review of studies from multiple countries found that people are generally distrustful of genomic research when private companies are involved.^26^ Similarly, focus groups of White inhabitants near Buffalo, NY reported concerns about the profit motives of pharmaceutical companies.^22^ Compared with the mostly urban participants in those studies, many of the patients we interviewed were concerned about both profit motives and government involvement. It is not clear whether skepticism of government research is unique to Mainers or is a temporal change related to contemporary politics in the United States. Regardless of these reported concerns, many participants report willingness to participate for altruistic reasons,^22,24,25^ or with their oncologist’s recommendation.^24,27,28^ Rural patients, who often have fewer educational and financial resources, are generally less knowledgeable of precision oncology research than their urban peers,^29^ and thus less likely to be aware of opportunities unless informed by their oncologist, making oncologist recommendations even more impactful.^30^ Other CMB sites serving rural populations may anticipate similar barriers. Future studies should keep these burdens in mind during research design to ensure adequate participation.

Although less burdensome than many therapeutic trials, biobanking studies that involve research-only biopsies—such as CMB—share many barriers previously identified for rural inhabitants: transportation, costs, medical procedures and associated risks, and patient effort.^27,31,32^ Oncologists and staff highlighted challenges around enrolling patients with fresh biopsies, especially when archival tissue was available. While research-only biopsies are optional for participating patients, they are necessary to CMB’s goal of understanding why cancer treatments lose efficacy over time.^1^ Patient reluctance around research-only biopsies agreed with past research establishing that patients are significantly less likely to agree to research-only biopsies compared with additional pass biopsies.^33,34^ However, in cases where patients are seeking new treatment options, they may see research participation as a means to that end, leading to greater tolerance of burdens and risk.^35-37^

Based on principles around “designing for the margins” in disability studies, it is our hope that addressing these barriers will make research more accessible for all populations served. Already these findings have informed interventions at MHCCN sites to boost CMB enrollment. A network-wide team was created to centrally manage CMB and other precision oncology studies. Team members are deployed to sites across the network to handle screening, enrollment, management of patients and biospecimen collection, removing the time and effort burden on smaller, rural-facing clinics. For CMB and other similar studies at MHCCN sites, the screening workflow was updated to allow for initial contact with the patient to come from the CRC rather than the physician. The treating physician is made aware of the patient’s potential eligibility and forthcoming patient contact. This allows for a much more direct and timely interaction with the patient and reduces burden on the physician. Beyond MHCCN, we have shared our results and recommendations to improve CMB at monthly meetings with the core CMB research team at NCI and other NCORP sites participating in the study.

At the time of publication, we are collaborating with the Cancer Resource Center of Western Maine to engage community members in designing an intervention to address misconceptions about research and encourage more participation by rural residents. If successful, we will pursue opportunities to expand the intervention to other Maine communities.

### Limitations

Interviews were focused on actual cancer research experiences when possible; however, because patient participants had not been invited to participate in CMB, they could not speak to actual barriers encountered in weighing CMB enrollment. Given the hypothetical nature of the interviews, patient participants may not have fully considered the question of CMB participation.

Participants were recruited from six counties, mostly located along Maine’s coast. Remote inland Maine counties were not represented. Greater rates of poverty and distance from health care sites in these unrepresented areas may have revealed additional barriers.

As our research focused on low income, rural inhabitants of Maine, we do not make claims about other populations. While most of our results were consistent with previous research, additional research is needed to determine the extent to which concerns and barriers we identified are present in other rural communities.

While appropriate for our research goals, our relatively small and nonrepresentative sample cannot be used to estimate the degree to which ideas heard in interviews are common in communities. Further research would be needed to estimate the popularity of different views. We are nevertheless confident that all barriers reported were common enough at the time of the study that triangulated sources confirmed them.

## Conclusions

This work highlights the education and support needs of low-income rural residents with cancer impacting awareness of, recruitment to, and retention in CMB and related research. Recognition of and engagement with enrollment and participation barriers is critical to equitable access to clinical studies by all served by our health system. This work is relevant not only to MHCCN sites but to other NCORP sites and cancer centers serving rural and low-income patients nationally.

## Supplementary Material

Supplement A_Clinician and Staff Interview Guide

Supplement B_Patient Interview Guide

Supplementary Data S1

Supplementary Data S2

## Figures and Tables

**Table 1.** Patient Participant Characteristics (*N* = 15)

*Variable*	*Measure* N (%)
Gender	
Male	6 (40)
Female	9 (60)
Age (years)	
<50	2 (13)
51–70	10 (67)
71+	3 (20)
Annual household income	
Not reported	6 (40)
< $25,000	5 (33)
$25–$49,999	1 (7)
$50–$75,000	3 (20)
Education	
Not reported	1 (7)
High school or below	6 (40)
Some college	4 (27)
College degree	3 (20)
Graduate degree	1 (7)
Insurance	
Medicare	8 (53)
Medicaid	4 (27)
Private	3 (20)
Previous clinical trial?	
Yes	1 (7)
No	14 (93)
Cancer type	
Bladder	1 (7)
Breast	7 (47)
Colorectal	2 (13)
Hematological	4 (27)
Lung	1 (7)
Metastatic?	
Yes	4 (27)
No	11 (72)

**Table 2.** Oncologist and CRC Participant Characteristics (*n* = 10)

*Variable*	*Measure* N (%)
Role	
Oncologist	6 (60)
CRC	4 (40)
Proportion patients low-income (estimate)	
25–44%	3 (30)
45–64%	3 (30)
65–84%	2 (20)
Unknown	2 (20)
Familiar with CMB?	
Yes	5 (50)
No	5 (50)
Exp. with precision oncology research?	
Yes	8 (80)
No	2 (20)

CMB, Cancer Moonshot Biobank; CRC, clinical research coordinators.

**Table 3.** Patient Perspectives on Cancer Research

Topic	Patient-reported [mis]understandings	Patient-reported concerns	Patient-reported reasons to participate
Cancer Research in general	All research involves a clinical trial/new drug	Requires time and effort Contact information could be sold Costs to participants Profit-motives of researchers Government involvement	Helps other people Participant may learn something
Clinical Trials	Involve taking experimental or untested drugs	Travel Health risks Requires changing treatment (even if it is working) May only be taking placebo	Path to better treatment (only if current treatment not working)
Genomic Research	Detects heritable cancer risk Involves treatment that “targets” cancer cells	None	Helps other people Learn about own cancer Better treatment (fewer side effects)
Biobanks	Place that stores tumors	Additional biopsies (involving travel and physical pain)	No concern about what happens to tumor Requires no effort from patient Helps other people

## Data Availability

Interview transcripts cannot be shared on a publicly accessible data repository because participants did not give permission. Limited data will be made available upon reasonable request from the corresponding author.
