# Supplementary material for: Potential Barriers to Participating in Cancer Moonshot Biobank for Low-Income Patients with Cancer of Rural Maine
Source: Biopreserv Biobank. Author manuscript; Available in PMC 2026 Mar 18. (PMC12997436; doi:10.1177/19475535251391568)
Supplement: Supplement A_Clinician and Staff Interview Guide [file NIHMS2146676-supplement-Supplement_A_Clinician_and_Staff_Interview_Guide.docx]

| **Moderators guide for ME-CGEC clinician and staff interviews**  We want to learn what you think about patients participating in tumor genomic studies and allowing their tissue to be sent to biobanks.  This interview is part of a research project, and your participation is purely voluntary. During the interview, I’ll ask you about your thoughts on tumor genomic studies and biobanking, including studies requiring sequential collection of biobanking samples throughout the patient’s treatment trajectory. We will discuss whether you would, or ever do, tell patients about these kinds of studies. Regardless of your opinion, your candid perspective is appreciated.  The interview will be recorded and later transcribed, and the recording itself will eventually be erased. You will not be identified as participating in this study. If you are quoted, you will only be identified as a clinician/staff. While we can try to protect your confidentiality, there is always a risk. After the interview, you will receive a $40 gift card in appreciation of your time. |
| --- |
| **Item** |
| 1. First I just need to collect some demographic information for reporting purposes:    1. How old are you?    2. With what gender do you identify?    3. With what racial or ethnic groups do you identify?    4. This research is predominantly about rural patients, particularly those with fewer financial resources. What proportion of the patients you see would likely fit that description? |
| 1. Next, can you tell me briefly of the experience you’ve had with your patients enrolling in tumor genome-targeted research? (probe exploratory/preclinical vs treatment clinical trials) |
| 1. What about tumor genome-targeted clinical trials? Tell me about that. 2. What about genomic research that involves biobanking? Any experience with that? Tell me about it. |
| 1. What do you think about patients participating in tumor genome-targeted research?    1. What do think is the value in patient participation? (Probe treatment clinical trials vs exploratory/preclinical studies)    2. Do you see any problems with it? (Alternative: What problems do you see with patients participating in these kinds of research?) (Probe: social/ethical concerns)    3. Is it always clear to you and patients everything that these kinds of studies involve? Tell me about that.       1. Can you think of examples of something that was surprising, or that you only learned after being involved in a study like this?    4. [Oncologists only] When patients participate in genome-targeted research, how does it impact your approach with a patient? |
| 1. How often do you talk to patients about tumor genome-targeted research studies?    1. [For oncologists] Do your patients ever ask you about genome-targeted clinical trials? Tell me about that.    2. What questions/concerns do your patients have about participating in tumor genome-targeted studies? [Probe: results impacting family members]    3. What are some of the things you’ve seen keep patients from enrolling in these kinds of studies? [Probe: practical/logistical issues, financial issues, misconceptions or concerns related to genetics, other?] |
| 1. Do you ever tell patients about biobanks that they can participate in?    1. What are some of the things you’ve seen keep patients from enrolling in biobanks specifically? [probe: practical issues, misunderstandings, other concerns?]    2. How would these barriers differ if collection of sequential samples is required? |
| 1. If [you want/the clinician wants] to inform a patient about a study or clinical trial for which they are eligible, how is that information given (e.g. pamphlets, research nurse/staff, directly from [you/the clinician])? |
| - 1. Why do you tend to use that mode of giving information? (What’s the advantage of doing that, what problems do you encounter with these different approaches?)   2. Is there a different approach you would prefer? If so, why not do that? |
| 1. In cases where you use a pamphlet or flyer, what kinds of things make these written materials more helpful?    1. Based on what you’ve seen, what kinds of things make them less helpful?    2. Are there particular kinds of questions patients tend to ask even after they’ve read them? Like what? |
| 1. What about for you? How do you typically learn about research studies and clinical trials? (Probes: What do you think about that? Is there a way you prefer?) |
| 1. Have you ever heard of the Cancer Moonshot Biobank project? [If so, what do you know about it?]    1. Briefly, the Cancer Moonshot Biobank collects biospecimens longitudinally, over the whole period of time that a person is getting cancer treatment and provides tumor genomic testing data to the provider and patient. The biospecimens and associated health information are also made available to researchers to study how cancer grows and changes in people, and to find new cancer treatments. Based on the sequential tumor genomic test results, some participants may be given the option of enrolling in clinical trials. [Add info on cancer type/stage]    2. What other information would you want to know about a project like this [before you would feel comfortable talking to your patients about it]? |
| 1. What would you think about a patient enrolling in CMB?    1. [Oncologists] Would you recommend a project like CMB to most of your patients? Is there a specific type of patient for which you would think it’s more appropriate?    2. What concerns would *you* have? |
| 1. [If not already discussed] What about the ongoing collection of biospecimens? What do you think about that aspect of CMB?    1. [Oncologists] If a patient of yours were enrolled in CMB, and you were getting longitudinal tumor genomic information, would that impact your approach with the patient? Tell me more about that.    2. Does it raise any additional concerns? |
| 1. [If oncologist is familiar with CMB] How would you explain a research study like Moonshot to a patient?    1. In your experience, what terms are most helpful / most confusing for patients?    2. What information do you think is most important for patients to understand before they participate in a study like this?    3. Based on past experiences for other studies, what concerns would you expect to hear from patients?    4. What about the ongoing collection of biospecimens? What concerns or challenges would you predict related to that? |
| 1. Do you think most of your patients would be interested in participating in the Cancer Moonshot Biobank? |
| 1. Why/Why not? 2. Based on your past experience, what are some reasons a patient might enroll? 3. What are some reasons patients might not? 4. If a patient wanted to enroll, what practical issues are there to consider? |
| 1. As mentioned, some patients who participate in CMB may learn they’re eligible for a tumor genome-targeted clinical trial. Would that be a factor in decisions to enroll? |
| 1. In your experience, what kinds of genome-targeted clinical trials are of most interest to patients? |
| 1. So the purpose of our study is to understand the kinds of things that make it difficult or unlikely for a patient to participate in tumor genome-targeted research. Can you think of anything else that we haven’t talked about which could be relevant? |
