# Supplementary material for: Potential Barriers to Participating in Cancer Moonshot Biobank for Low-Income Patients with Cancer of Rural Maine
Source: Biopreserv Biobank. Author manuscript; Available in PMC 2026 Mar 18. (PMC12997436; doi:10.1177/19475535251391568)
Supplement: Supplement B_Patient Interview Guide [file NIHMS2146676-supplement-Supplement_B_Patient_Interview_Guide.docx]

| **Interview guide for Patients**  The reason we’re doing this interview is to help figure out why some people decide to be in cancer research and some don’t.  In the interview today, I want to learn what *you think* about participating in certain kinds of cancer research. To be clear I’m only going to be asking questions. I’m not asking you to actually enroll in cancer research.  This interview is for research only. You don’t have to participate in the interview. You can skip questions if you want, and we can end the interview early if you want. We will only continue if you want to.  During the interview, I’ll ask you about experiences you’ve had with research, and your experiences with cancer, especially as it relates to participating in research. A lot of my questions will be hypothetical—about what *might* happen in a certain situation. In the interview I’m going to ask about “tumor genomics” and “biobanks.” It’s fine if you don’t know what those mean.  The interview will be recorded and later transcribed, and the recording itself will eventually be erased. You will not be identified as participating in this study. If you are quoted, you will only be identified as a patient. We will leave out any information that someone could use to figure out who you are. After the interview, you will receive a $40 gift card in appreciation of your time.  Before we start, I have a few demographic questions. (ask Q 14 or do Redcap survey). |
| --- |
| **Item** |
| 1. First, can you give me an idea about where you are in your journey with cancer? |
| 1. How long ago did you get your diagnosis? 2. And what is your current diagnosis? Do you know what stage the cancer is? |
| 1. Have you ever been involved in cancer research? Tell me more about that.    1. [If not] What about other kinds of research?    2. Why did you decide to participate in that?    3. What did you think about the experience?    4. Did anything about it surprise you?    5. Was there anything about being in that study that was hard for you?    6. If you had it to do over, would you participate again? Why/why not? |
| 1. Where did you hear about that research? / Where would you expect to hear about research you can participate in? [Probe: oncologist vs other sources]    1. If you were being told about a cancer research study, would you want someone to explain it to you, or would you rather have something to read like a brochure or a website? Why? |
| 1. Have you ever heard the term “cancer genomics”?    1. What have you heard about it?    2. When you hear “cancer genomics” what do you think about? |
| 1. I have a little explanation here for what scientists mean by “cancer genomics” : Cancer genomics is where researchers compare the genes in normal cells in your body with the genes of cancer cells in your body. Those differences may be useful for figuring out how the cancer will respond to different kinds of treatment.    1. Does that explanation make sense?    2. What questions or concerns do you have about it? |
| 1. Participating in cancer genomics research usually involves sending either your blood or parts of your tumor to a laboratory to read the genetic code of the tumor.    1. What questions or concerns would you have about participating in cancer genomics research?    2. Why do you think a person who has cancer would want to participate in genomic research?    3. Thinking about where you are with cancer, if you were asked to participate in a cancer genomics study, would you consider participating? |
| 1. When patients participate in genomics research they sometimes find out about different drugs that could be good for treating their cancer. This doesn’t always happen, but it’s possible. Do you think the possibility of new treatment options would be enough of a reason for you to want to participate?    1. [If so] What would you hope for by getting into a study like this? |
| 1. Sometimes, patients who participate in cancer genomics find out that they are eligible for clinical trials. What does the term “clinical trial” mean to you?    1. Clinical trials are like experiments to test new drugs and see if they’re better than existing drugs. What do you think about that?    2. If they were testing a new cancer drug and you could be part of that test, would you want to? What questions or concerns would you have? [Probe factors that affect the decision]    3. Would the possibility of being in a clinical trial be enough of a reason to participate in cancer genomics research? Why/why not? |
| 1. What if there was no clinical benefit, meaning it wouldn’t give you new options? Would you or other people with cancer still want to participate in a study like that? Why/why not?    1. One reason I’ve heard is that this research could help discover better treatments for other patients in the future, so it’s a way to help other people, but doesn’t help you personally. What do you think about that? Would that be a good enough reason for you to participate? |
| 1. Have you ever heard of tumor biobanking? |
| - 1. What does that mean to you? When you hear “tumor biobanking” what does that make you think about? |
| 1. I have a little explanation of what scientists mean by biobanking: Biobanking is when tissue samples, like blood or bits of a tumor, are collected from an individual and sent to a place where it is stored and used for medical research.    1. What does taking a sample of your tissue or blood mean to you?    2. What questions do you have about researchers sending your tissue or blood and to a biobank and using it for research?    3. Would it matter who the researchers worked for? [probe corporation, government, non-profit, etc.] |
| (11) Some biobanking studies involve ongoing tissue collection. Instead of collecting it only once, they would collect it multiple times, like any time you have a biopsy. What do you think about that?  a. If you were asked to participate in a biobank that asked for ongoing tissue collection, what questions would you have?  b. If you were going to participate in something like that, what practical issues do you think you might run into? (Probe: driving to location, taking off work, side effects of tissue collection) |
| 1. We’re getting close to the end of the interview. As I mentioned before, the purpose of our study is to understand the kinds of things that make it difficult or unlikely for a patient to participate in cancer genomics and biobanking research. Can you think of anything else that we haven’t talked about which could be relevant? |
| 1. Finally I have some basic demographic questions. As a reminder you can skip any of these if you’d prefer:    1. How old are you? (range by decade)    2. With what gender do you identify?    3. With what racial or ethnic groups do you identify?    4. What county do you live in?    5. What’s your household income? (range by $25,000)    6. What’s the highest level of education you’ve reached?    7. How many people are in your household?    8. What’s your insurance status? (private/commercial, Medicare, Medicare+supplement Mainecare, none    9. Cancer type    10. Cancer stage    11. Time since last cancer treatment    12. Ever participated in clinical trial? |
